# Advanced gastric cancer with abdominal wall invasion treated with curative resection after chemotherapy: a case report

**DOI:** 10.1186/s13256-021-02820-7

**Published:** 2021-05-09

**Authors:** Naohiko Nakamura, Shinichi Kinami, Jun Fujita, Daisuke Kaida, Yasuto Tomita, Takashi Miyata, Hideto Fujita, Nobuhiko Ueda, Hiroyuki Takamura

**Affiliations:** Department of Surgical Oncology, Kanazawa Medical University, 1-1 Daigaku, Uchinada, Kahoku, Ishikawa 920-0293 Japan

**Keywords:** Gastric cancer, T4b, Abdominal wall invasion, Gastrectomy, Case report

## Abstract

**Introduction:**

In patients with gastric cancer, 6–27% of patients are diagnosed with T4b disease that invades adjacent organs, and curative resection can improve the prognosis of these patients.

**Case presentation:**

A 70-year-old Japanese man presented with an abdominal tumor and was diagnosed with advanced gastric cancer (L-Circ type 3 T4b N2 M0 H0 stage IVA, based on the 15th edition of the Japanese Classification of Gastric Carcinoma) with extensive abdominal wall invasion. We performed open gastrojejunal bypass for gastric obstruction and initiated a chemotherapeutic regimen comprising S-1 (120 mg/day) and oxaliplatin (100 mg/m^2^). Upper gastrointestinal endoscopy performed after the administration of six courses of the S-1 and oxaliplatin regimen revealed a persistent primary lower gastric wall lesion; however, the diameter of the abdominal wall invasion and metastatic lymph nodes was significantly reduced, in addition to decreased serum carcinoembryonic antigen and carbohydrate antigen 19-9 levels. Subsequently, the patient underwent distal gastrectomy with D2 lymphadenectomy combined with transverse colon and abdominal wall resection. We performed radical en bloc resection and achieved a tumor-free resection margin. Simple abdominal wall closure was performed without mesh or musculocutaneous flap placement. Histopathological examination of the resected tumor specimen showed direct invasion of the mesocolon and rectus abdominis muscle. The patient was postoperatively diagnosed with L Gre-Ant type5 T4b (SI: rectus abdominis muscle) N2 PM0 DM0 Stage IIIA R0 Grade 2a gastric cancer based on histopathological findings and received S-1 as adjuvant chemotherapy, 2 months postoperatively. No recurrence was detected 6 months postoperatively.

**Conclusions:**

We report a case of advanced gastric cancer with extensive abdominal wall invasion that was successfully treated with gastrectomy combined with resection of adjacent organs showing tumor invasion after effective systemic chemotherapy. A therapeutic approach comprising curative surgery combined with perioperative chemotherapy is useful in patients with T4b gastric cancer.

## Background

Gastric cancer (GC) is the fifth most common malignancy and the third most common cause of cancer mortality worldwide [1]. Although early GC is curable, advanced-stage disease is associated with poor survival, and curative treatment consists of gastrectomy with perioperative chemotherapy [2, 3]. Based on the 8th edition of the International Union Against Cancer TNM classification and the 15th edition of the Japanese Classification of Gastric Carcinoma, advanced GC that invades adjacent organs is classified as T4b GC [4, 5]. Notably, 6–27% of patients with GC are diagnosed with T4b disease [6–11], and curative resection can improve the prognosis of these patients [6–9]. Curative resection is indicated in ≤ 50% of patients with T4b GC because these patients invariably present with peritoneal dissemination and distant metastasis that frequently complicate T4b GC [12–14]. Therefore, accurate diagnosis of progressive disease and preoperative administration of effective chemotherapy are important to achieve curative resection in patients with T4b GC.

We present a case of advanced GC with extensive abdominal wall invasion that was successfully treated with gastrectomy combined with adjacent organ resection after effective systemic chemotherapy.

## Case presentations

**Fig. 1:**
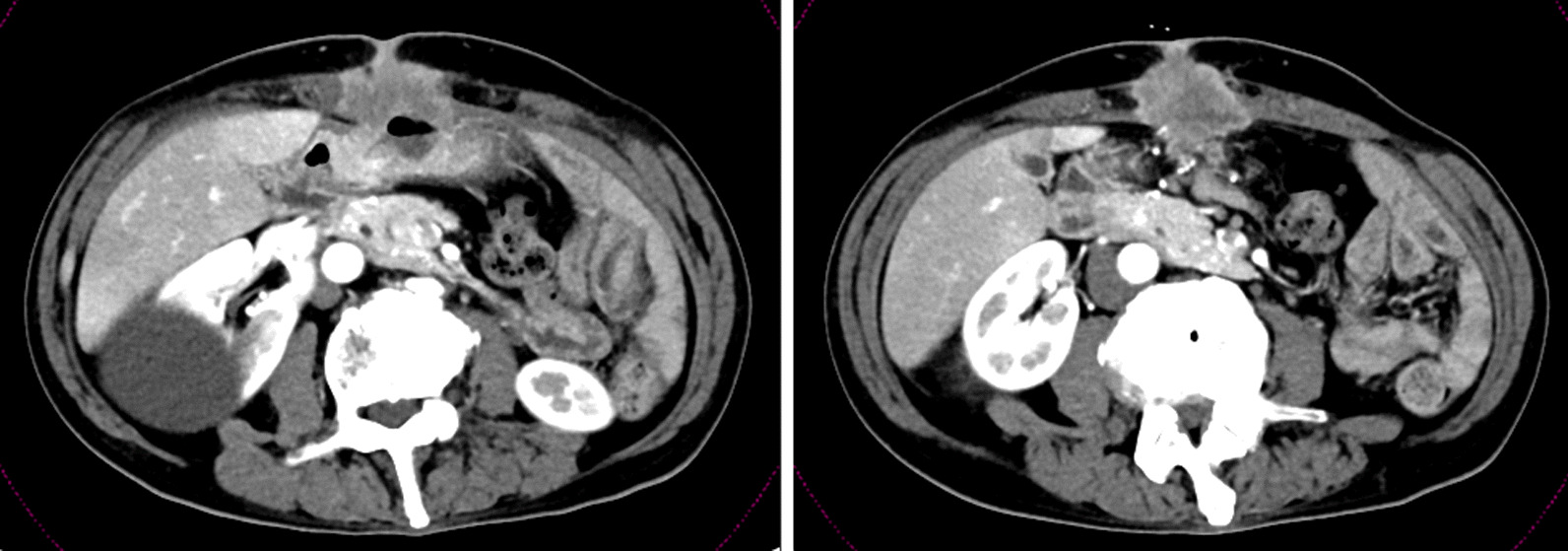
Pretreatment contrast-enhanced computed tomography scan showing the primary gastric tumor with massive invasion of the abdominal wall and enlarged peritumoral lymph nodes

**Fig. 2:**
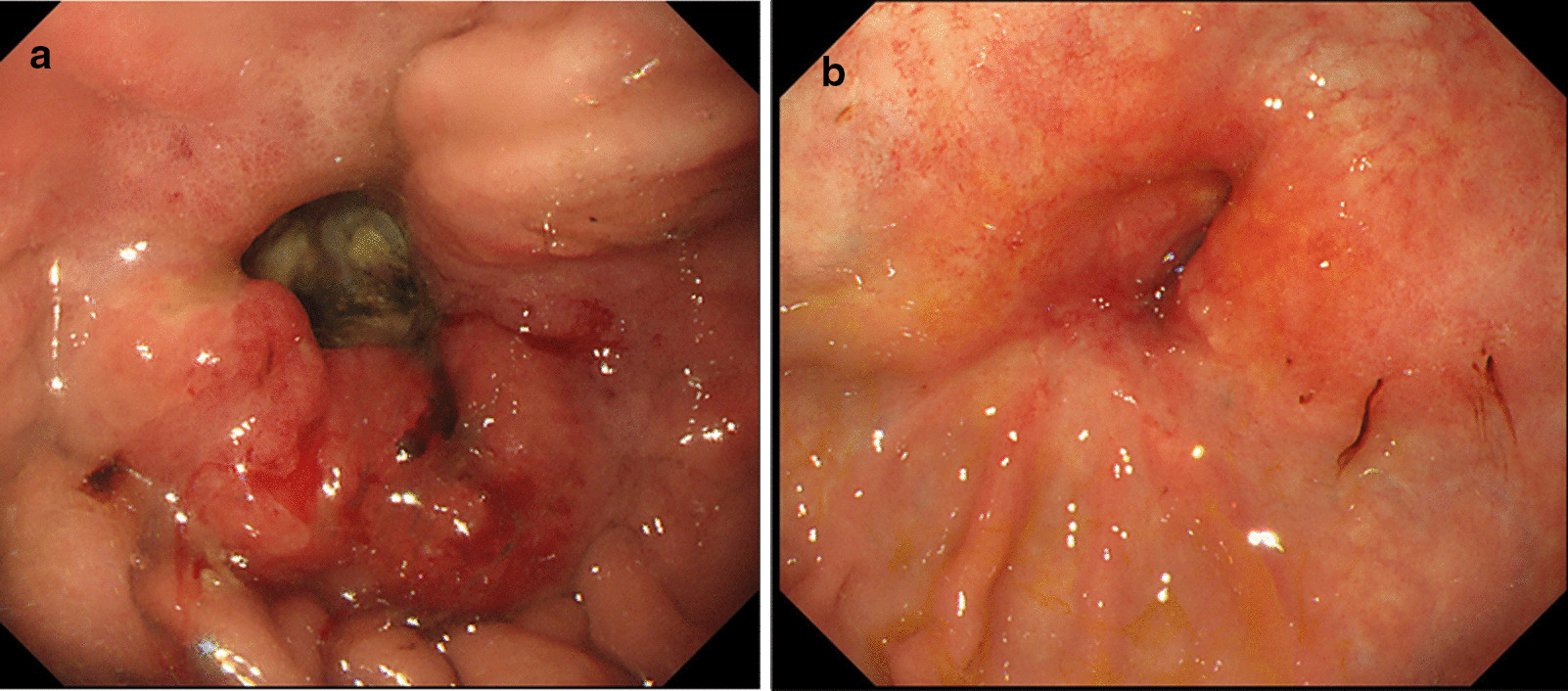
Upper gastrointestinal endoscopy image obtained before treatment (**a**) and image obtained after chemotherapy (**b**) showing the following findings: **a** Borrmann type III tumor with stenosis observed at the lower gastric wall. **b** A persistent primary lesion visualized at the lower gastric wall; however, the lesion has regressed in size

**Fig. 3:**
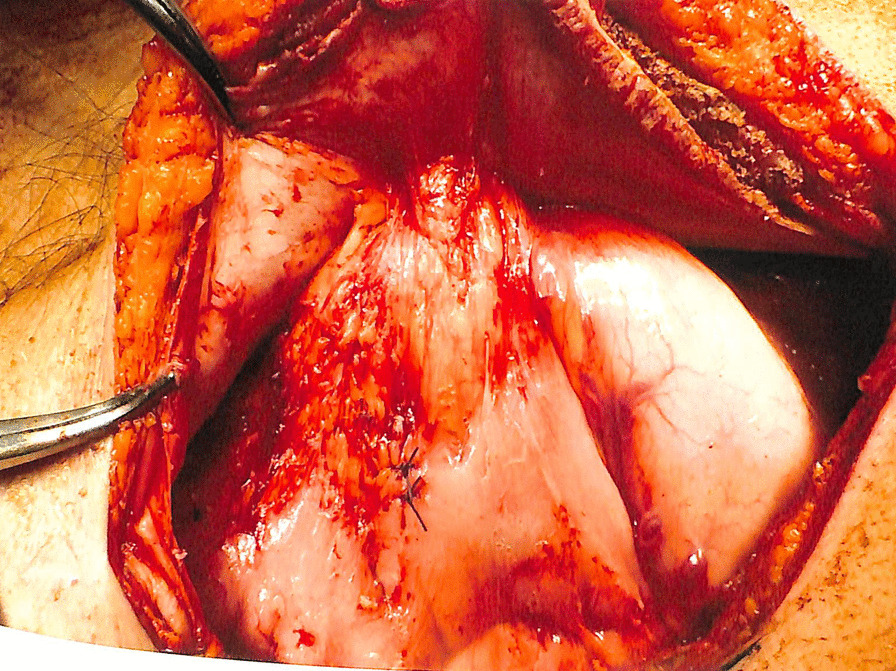
Intraoperative finding during open gastrojejunal bypass

**Fig. 4:**
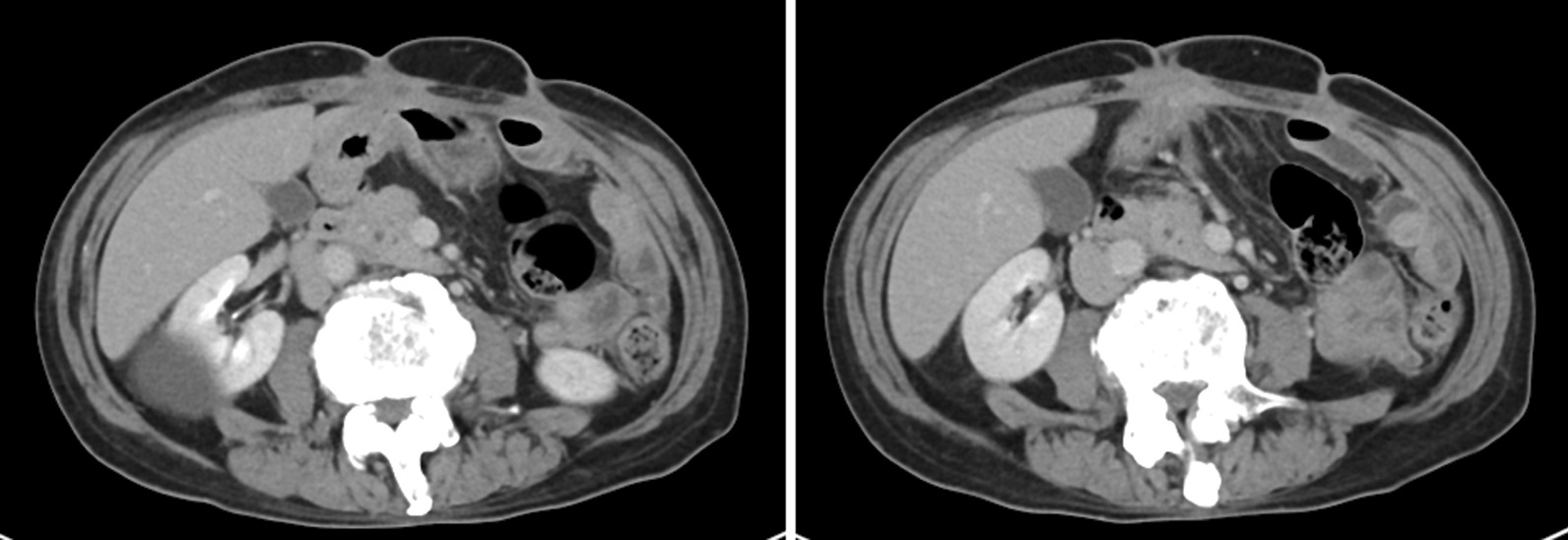
Contrast-enhanced computed tomography scan obtained after six courses of the SOX regimen showing significant reduction in the diameter of the abdominal wall invasion and metastatic lymph nodes. SOX regimen: chemotherapeutic regimen comprising S-1 (120 mg/day) and oxaliplatin (100 mg/m^2^)

A 70-year-old Japanese man (height 178 cm, weight 54.0 kg, and Eastern Cooperative Oncology Group Performance Status score 0) presented with an abdominal tumor and suspected advanced GC. He had previously undergone an operation for ileus, and his family history was not significant. A contrast-enhanced computed tomography scan (eCT) revealed extensive abdominal wall invasion by the main gastric tumor in addition to enlarged peritumoral lymph nodes; however, no lung and liver metastases were detected (Fig. 1). Upper gastrointestinal endoscopy revealed a circumferential Borrmann type III tumor at the lower gastric wall with tumor-induced stenotic obstruction (Fig. 2a). Biopsy of the gastric tumor revealed a moderately differentiated tubular adenocarcinoma. Immunohistochemical evaluation showed a human epidermal growth factor receptor 2-negative lesion, and the patient was clinically diagnosed with L-Circ type 3 T4b N2 M0 H0 stage IVA GC (based on the 15th edition of the Japanese Classification of Gastric Carcinoma [5]). We performed open gastrojejunal bypass for gastric obstruction and confirmed extensive abdominal wall invasion of the tumor and absence of peritoneal dissemination (Fig. 3). Then, we initiated the SOX chemotherapeutic regimen comprising S-1 (120 mg/day, day 1 to day 14) and oxaliplatin (100 mg/m^2^, every 21 days). Although neutropenia (Grade 3, based on the National Cancer Institute Common Terminology Criteria for Adverse Events version 3.0) occurred as an adverse event associated with SOX therapy, the diameter of the abdominal wall invasion and metastatic lymph nodes was reduced after the administration of four courses of the SOX regimen. Therefore, we administered six courses of the SOX regimen. Upper gastrointestinal endoscopy revealed a persistent primary lesion of the lower gastric wall (Fig. 2b); however, the diameter of the abdominal wall invasion and metastatic lymph nodes was significantly reduced in addition to decreased serum carcinoembryonic antigen and cancer antigen 19-9 levels (Fig. 4). Notably, eCT performed after six courses of the SOX regimen revealed a partial response to therapy based on The Response Evaluation Criteria in Solid Tumors, version 1.1 guidelines. Subsequently, we performed distal gastrectomy with D2 lymphadenectomy combined with transverse colon and abdominal wall resection because we observed direct tumor invasion of the mesocolon and rectus abdominis muscle. We performed radical en bloc resection and achieved a tumor-free resection margin. Although the tumor invaded the rectus abdominis muscle for which we performed abdominal wall resection, the abdominal wall defect was closed using simple sutures without mesh or musculocutaneous flap placement. Based on histopathological examination, the patient was postoperatively diagnosed with L Gre-Ant yp-TRype5 T4b (SI: rectus abdominis muscle) N2 PM0 DM0 Stage IIIA R0 Grade 2a GC (according to the 15th edition of the Japanese Classification of Gastric Carcinoma [5]) and received S-1 as adjuvant chemotherapy, 2 months postoperatively. No recurrence was detected on 6-month postoperative eCT.

## Discussion and conclusions

Curative resection is the mainstay of treatment for patients with T4b GC [6–9]. However, this treatment strategy remains controversial because T4b GC is a heterogeneous and multifactorial disease. In this case, we administered systemic chemotherapy before surgical resection to ensure curative resection by minimizing invasion of adjacent organs, particularly the abdominal wall. Reportedly, the 5-year survival rate in patients with T4b GC who undergo curative resection is 20–32%, which is significantly higher than the survival rate after noncurative resection (7–9%) [13, 14]. Studies have reported that patients with distant metastases showed poor prognosis even after undergoing resection of adjacent organs showing tumor invasion [12]. The pancreas, mesocolon, liver, transverse colon, adrenal glands, and spleen are most commonly invaded (based on macroscopic appearance) [10]. In this case, the tumor macroscopically invaded the mesocolon and abdominal wall. Although curative resection is possible without administration of preoperative chemotherapy, an effective response to preoperative chemotherapy contributes to successful radical en bloc resection and also improves postoperative prognosis. In this patient, the final histopathological findings revealed lymph node metastasis (stage N2). Previous studies have reported a correlation between advanced stage lymph node disease and poor prognosis after curative resection in patients with T4b GC [15, 16]. Therefore, preoperative chemotherapy may be important for patients with T4b GC with advanced-stage lymph node disease. Moreover, administration of preoperative chemotherapy significantly reduced the diameter of the abdominal wall invasion; therefore, the abdominal wall defect could be closed with simple sutures without mesh or musculocutaneous flap placement. Tumor stage, expected survival, and postresection quality of life should be carefully evaluated for optimal planning to minimize the extent of surgery.

In conclusion, we report a case of advanced GC with extensive abdominal wall invasion that was successfully treated with gastrectomy combined with resection of adjacent organs showing tumor invasion after effective systemic chemotherapy. In our view, a therapeutic approach comprising curative surgery combined with perioperative chemotherapy is useful in patients with T4b GC.

## Data Availability

All data are available without restriction. Researchers can obtain data by contacting the corresponding author.

## Abbreviations

GC: Gastric cancer
TNM: Tumor node metastasis
eCT: Contrast-enhanced computed tomography scan
SOX: Chemotherapy combining S-1 and oxaliplatin
